# Spectrum of Genetic Variants in a Cohort of 37 Laterality Defect Cases

**DOI:** 10.3389/fgene.2022.861236

**Published:** 2022-04-13

**Authors:** Dinu Antony, Elif Gulec Yilmaz, Alper Gezdirici, Lennart Slagter, Zeineb Bakey, Helen Bornaun, Ibrahim Cansaran Tanidir, Tran Van Dinh, Han G. Brunner, Peter Walentek, Sebastian J. Arnold, Rolf Backofen, Miriam Schmidts

**Affiliations:** ^1^ Genome Research Division, Human Genetics Department, Radboud University Medical Center and Radboud Institute for Molecular Life Sciences, Nijmegen, Netherlands; ^2^ Center for Pediatrics and Adolescent Medicine, University Hospital Freiburg, Faculty of Medicine, Freiburg, Germany; ^3^ Department of Medical Genetics, University of Health Sciences, Istanbul Kanuni Sultan Suleyman Training and Research Hospital, Istanbul, Turkey; ^4^ Department of Pediatric Cardiology, University of Health Sciences, Istanbul Kanuni Sultan Suleyman Training and Research Hospital, Istanbul, Turkey; ^5^ Department of Pediatric Cardiology, Basaksehir Cam and Sakura City Hospital, Istanbul, Turkey; ^6^ Bioinformatics Group, Department of Computer Science, University of Freiburg, Freiburg, Germany; ^7^ Maastricht University Medical Center and GROW School of Oncology and Development, Maastricht University, Maastricht, Netherlands; ^8^ Renal Division, Department of Medicine, University Hospital Freiburg, Faculty of Medicine, University of Freiburg, Freiburg, Germany; ^9^ CIBSS- Centre for Integrative Biological Signalling Studies, University of Freiburg, Freiburg, Germany; ^10^ Institute of Experimental and Clinical Pharmacology and Toxicology, Faculty of Medicine, University of Freiburg, Freiburg, Germany

**Keywords:** laterality defect, situs inversus, exome, cilium, primary ciliary dyskinesia, dynein, PIFO, PKD1L1

## Abstract

Laterality defects are defined by the perturbed left–right arrangement of organs in the body, occurring in a syndromal or isolated fashion. In humans, primary ciliary dyskinesia (PCD) is a frequent underlying condition of defective left–right patterning, where ciliary motility defects also result in reduced airway clearance, frequent respiratory infections, and infertility. Non-motile cilia dysfunction and dysfunction of non-ciliary genes can also result in disturbances of the left–right body axis. Despite long-lasting genetic research, identification of gene mutations responsible for left–right patterning has remained surprisingly low. Here, we used whole-exome sequencing with Copy Number Variation (CNV) analysis to delineate the underlying molecular cause in 35 mainly consanguineous families with laterality defects. We identified causative gene variants in 14 families with a majority of mutations detected in genes previously associated with PCD, including two small homozygous CNVs. None of the patients were previously clinically diagnosed with PCD, underlining the importance of genetic diagnostics for PCD diagnosis and adequate clinical management. Identified variants in non-PCD-associated genes included variants in *PKD1L1* and *PIFO*, suggesting that dysfunction of these genes results in laterality defects in humans. Furthermore, we detected candidate variants in *GJA1* and *ACVR2B* possibly associated with situs inversus. The low mutation detection rate of this study, in line with other previously published studies, points toward the possibility of non-coding genetic variants, putative genetic mosaicism, epigenetic, or environmental effects promoting laterality defects.

## Introduction

**FIGURE 1:**
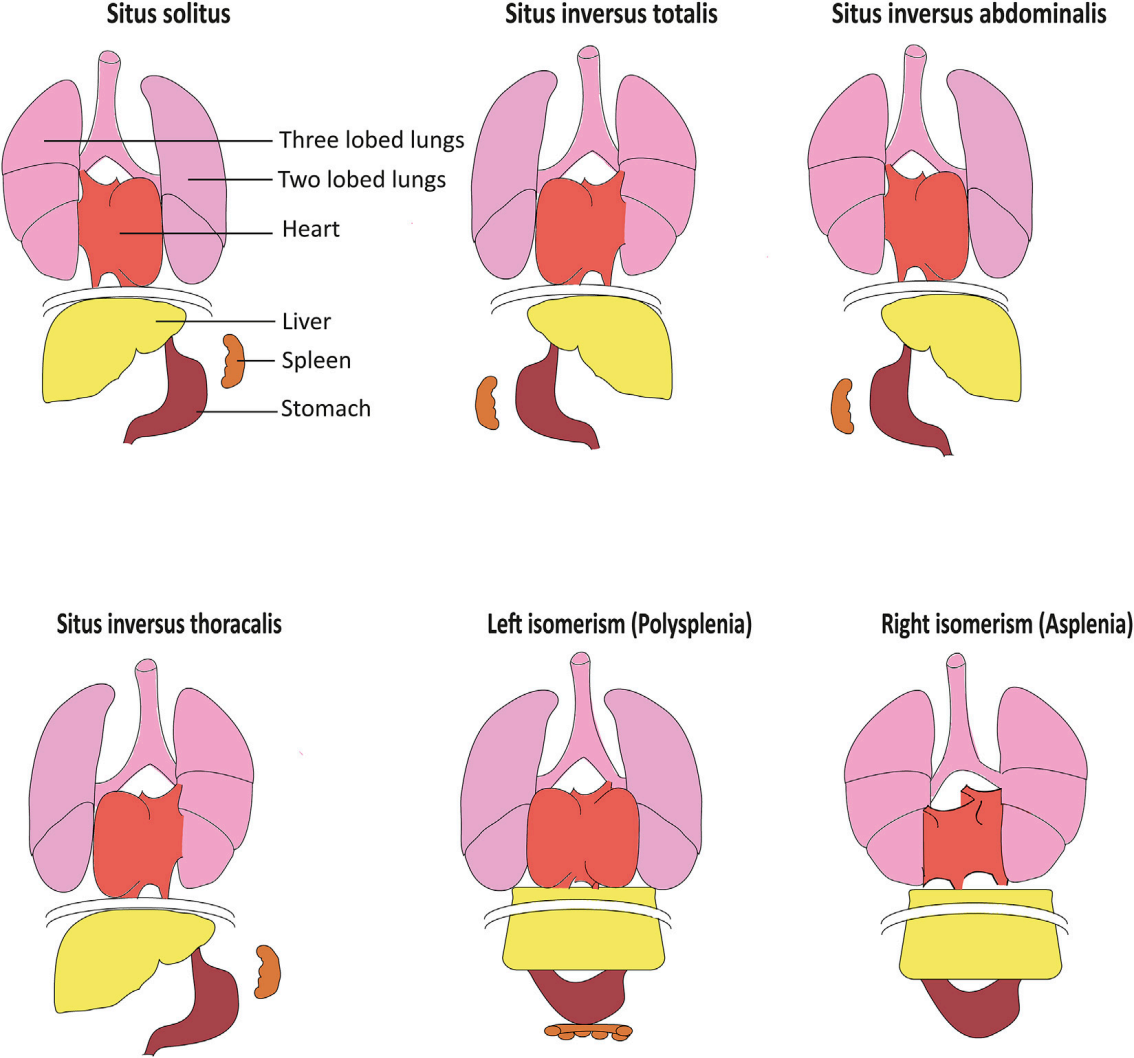
Organ arrangement patterns in humans. While situs solitus represents the most frequent left–right organ arrangement, situs inversus represents the corresponding mirror image. In situs inversus thoracalis and situs inversus abdominalis, left–right patterning is inversed solely above or below the diaphragm. Left isomerism often results in polysplenia while accordingly, right isomerism can cause asplenia. Isomerism often also causes severe structural heart defects including defective arrangement of the great blood vessels, adapted from Fliegauf et al. (2007).

Laterality defects in humans occur with a frequency of about 1:10,000 (Lin et al., 2014). *Situs inversus totalis* presents with a complete mirror image of normal organ asymmetry throughout the body. In contrast, in *situs inversus thoracalis* or *abdominalis* (heterotaxy), only a subset of organs is affected. While *situs inversus* usually does not impair organ function, this is different in left or right isomerism or situs ambiguous, accounting for up to 5% of all laterality defect cases. Heterotaxy is commonly associated with complex heart defects such as transposition of the great arteries can significantly affect the quality of life and life expectancy (Lin et al., 2014). Furthermore, polysplenia can be observed with left isomerism, while right isomerism is associated with asplenia and increased risk for serious infections (Fliegauf et al., 2007) (Figure 1)

**FIGURE 2:**
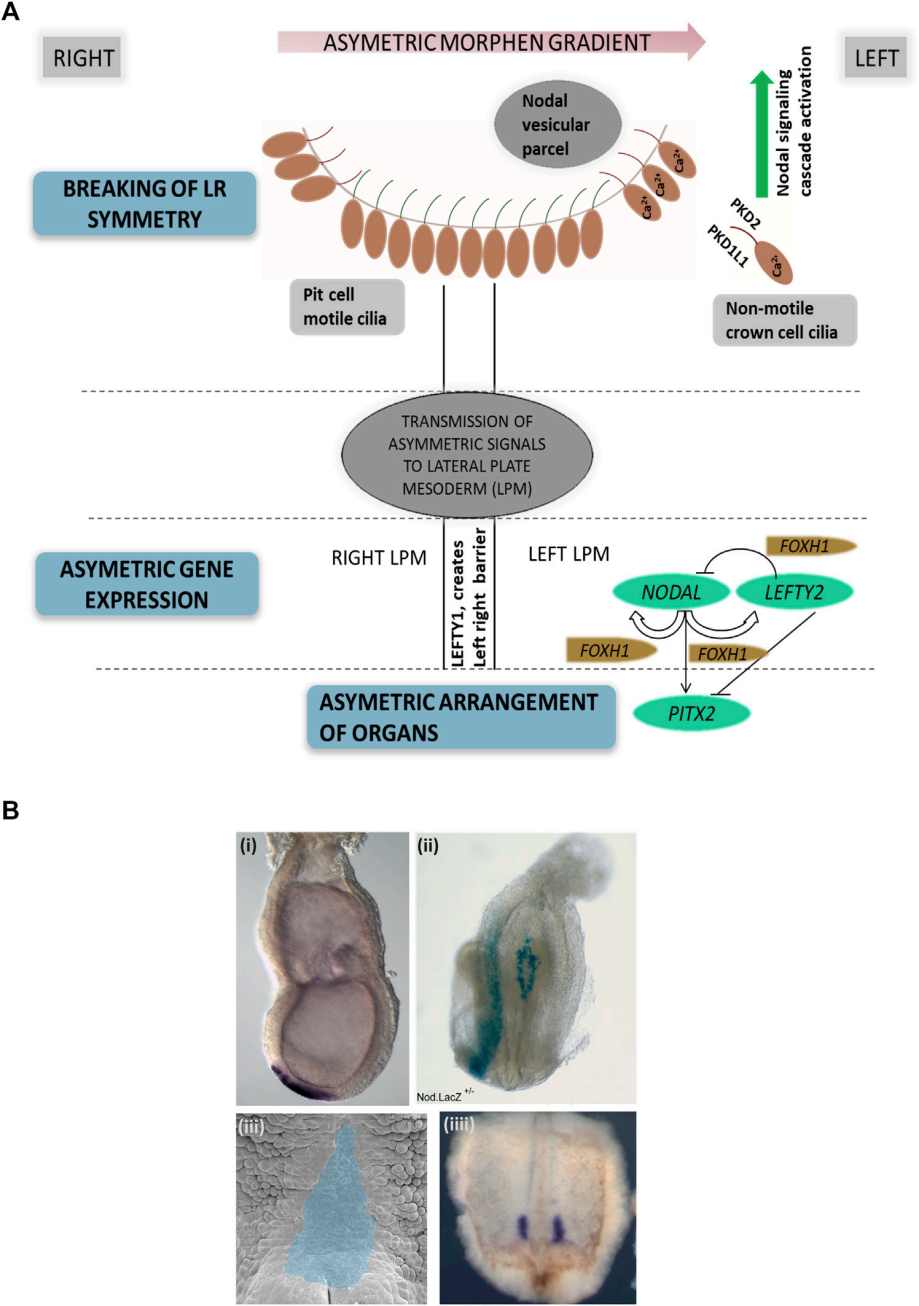
Left–right asymmetry in mammals. **(A)** Simplified representation of left–right patterning initiation in mammals. Breaking of left–right asymmetry is initiated by motile cilia in the embryonic node generating a leftward fluid flow followed by signal propagation by non-motile cilia. Here, PKD2 and PKD1L1 channels at the ciliary membrane enable calcium currents, resulting in elevated Ca2+ concentrations on the left side of the node. Node asymmetry is then propgated to the lateral plate mesoderm, which in turn causes NODAL expression in the left lateral plate mesoderm, followed by PITX2 expression. LEFTY2 acts as a feedback inhibitor restricting the range of nodal signaling. LEFTY1 helps in maintaining the midline barrier. **(B)** Examples of left–right organizing centers in vertebrates. (i) *In situ* hybridization visualizing the embryonic node (Shh, purple), e7.5 mouse embryo; (ii) Nodal.LacZ reporter expression (blue) marking the embryonic node and the lateral left plate mesoderm in an E8.5 mouse embryo; (iii) *Xenopus* embryo stage 17 archenteron roof in ventral perspective (pseudocolored in blue) where cilia-mediated leftward flow initiates left–right asymmetry; (iiii) *Xenopus* embryo stage 17 with nodal expression marked in purple (*in situ* hybridization).

Left–right body asymmetry in vertebrates is determined very early in embryonic development, such as embryonic day 7.5 in mice (Marszalek et al., 1999) and 6–12 somite stage in zebrafish (Kawakami et al., 2005). In mammals, the so-called embryonic node is a transiently present structure which exhibits motile 9 + 0 monocilia and non-motile 9 + 0 monocilia (Figures 2A,B). Both types of cilia are considered essential for left–right patterning as functional defects of both motile and non-motile cilia can result in laterality defects in vertebrates (Pennekamp et al., 2015). Dysfunction of genes encoding for proteins essential for the ciliary motility apparatus such as dynein arm components results in left–right patterning defects in humans (Leigh et al., 2019) and mice (Tan et al., 2007), most often combined with recurrent respiratory tract infections due to impaired mucociliary clearance and often infertility (Fliegauf et al., 2007). This combination of laterality defects, bronchiectasis, and sinusitis is also referred to as Kartagener syndrome, observed in approximately 50% of all primary ciliary dyskinesia (PCD) cases (Knowles et al., 2016) (Supplementary Table S1). Of note, mutations in genes encoding for proteins associated with the central pair microtubules and radial spokes result in PCD but do not cause situs abnormalities due to the 9 + 0 ciliary ultrastructure in the node compared to a 9 + 2 ultrastructure of motile airway cilia (Knowles et al., 2016). This includes mutations in *RSPH4A*, *HYDIN*, *MCIDAS*, *RSPH9*, *RPGR*, *CCDC65*, *RSPH1*, *CCDC164*, and *STK36* (Edelbusch et al., 2017). Further, mutations in CCNO (Wallmeier et al., 2014) and MCIDAS (Boon et al., 2014) cause mucociliary clearance disorders without laterality disturbances. Likewise, mutations in genes encoding for ciliary proteins unrelated to motility in humans and mice can also result in a disturbed left–right body axis, including *PKD2* (Pennekamp et al., 2002; Bataille et al., 2011), *INVERSIN* (Simons et al., 2005), *NPHP3* (Olbrich et al., 2003), and *PKD1L1* (Field et al., 2011; Vetrini et al., 2016). These mutations are usually combined with additional developmental defects such as renal-hepatic dysplasia, polycystic kidney disease, nephronophthisis, chondrodysplasias, brain malformation, or retinal degeneration (Fliegauf et al., 2007; Deng et al., 2015) (Supplementary Table S1).

Normal motile cilia function is crucial for generating a leftward flow within the embryonic node in mammals (Nonaka et al., 1998), initiating cell signaling pathways driving the establishment of body laterality. The “two-cilia hypothesis” proposes that a leftward flow generated by motile node pit cell cilia is sensed by non-motile node crown cell cilia which causes calcium influx determining the left side of the embryonic node (McGrath et al., 2003). A second model proposes that the leftward nodal flow results in a morphogen gradient, including vesicular particles being transported toward the left side of the node where they release their content, creating a morphogen gradient (Tanaka et al., 2005) (Figure 2A)**.** The definition of the left side of the embryonic node subsequently leads to the activation of the TGFβ-superfamily components, namely, *NODAL* and *LEFTY2*. The nodal antagonist *LEFTY2* prevents the activation of *NODAL* on the right side of the lateral plate mesoderm (Juan and Hamada, 2001; Shiratori and Hamada, 2006). On the left side, *NODAL* consecutively induces *PITX2* expression on the left side of the lateral plate mesoderm, enabling asymmetric heart, lung, and spleen development (Norris, 2012). Midline *LEFTY1* expression confines *NODAL*, *LEFTY2*, and *PITX2* expression to the left side as *LEFTY1* null mice display bilateral expression of *NODAL*, *LEFTY2*, and *PITX2* (Meno et al., 1998; Capdevila et al., 2000), (Grimes and Burdine, 2017) (Figure 2A). It is therefore not surprising that nodal dysfunction in mammals causes laterality defects (Mohapatra et al., 2009).

In addition to nodal signaling, Bmp, Notch, Hedgehog, and Fgf signaling also play important roles for left–right establishment (Sempou and Khokha, 2019). In humans, mutations in *ZIC3* (Gebbia et al., 1997), the TGF-beta signaling molecule *GDF1* (Karkera et al., 2007) or *ACVR2B* (Kosaki et al., 1999; Ma et al., 2012), and the metalloprotease *MMP21* (Guimier et al., 2015) have been associated with left–right patterning defects. However, past genetic studies investigating laterality defects in humans have had a surprisingly low yield of causative mutations, leaving up to 90% of cases genetically unexplained (Table 1). As progress has since been made with the identification of novel laterality defect genes and some of the previous studies did not use exome sequencing but targeted Sanger sequencing or gene panel sequencing, we performed an exome-based study in a cohort of 37 Turkish individuals with laterality defects to determine the rate of mutations in known disease genes and to identify novel disease-causing alleles.

**TABLE 1:** Summary of previously published studies describing genetic screening in cohorts with laterality defects. Search terms included “laterality defect,” “heterotaxy,” “situs inversus,” “primary ciliary dyskinesia,” and “genetic” or “mutation.” Success rates were stated as presented in the manuscripts or calculated from the results presented in the publications.

Cohort phenotype	Ethnicity	Variant identification methods used	Success rate	Publication
Congenital heart disease	European 2,063, African American 189, East Asian 36, South Asian 136, and Mexican 280, Other 167	Whole-exome sequencing of probands (2,871 cases)	10.1% of 2,871 cases.	Jin et al. (2017)
Fetuses with congenital heart defects and/or heterotaxy and no cytogenetic anomalies	Ethnicity information not available, two parents were consanguineous	Targeted NGS panel	10/80 fetuses (12.5%)	Liu et al. (2020)
Abnormal atrial situs (atrial isomerism or atrial situs inversus)	Arabic	Whole-exome sequencing	17/30 cases (56.6%)	Bolkier et al. (2021)
Transposition of the great arteries not associated with other situs anomalies	Italian	Coding sequence analysis of *ZIC3, ACVR2B, LEFTYA, CFC1, NODAL, NKX2.5, CRELD1, GATA4, GDF1*, and *FOXH1*	2/7 families (28.5%)	De Luca et al. (2010)
Heterotaxy patients with cardiac manifestations	Caucasian 21, Hispanic 11, African American 9, Asian and Southeast Asian 3, and other 3	Coding sequence analysis of *ZIC3, LEFTYA, ACVR2B*, and *CFC1*	4/47 cases (8.5%)	Ma et al. (2012)
Congenital heart disease and heterotaxy	Clinical Genetics, Maastricht University Medical Center, Maastricht, Netherlands	Screened only for *ZIC3* variant	6/348 cases (1.7%)	Paulussen et al. (2016)
	Ethnicity not mentioned			
Sporadic heterotaxy patients with congenital heart defects	Chinese	Affymetrix CytoScan HD microarray and real-time polymerase chain reaction	19 rare CNVS in 63 cases (30.1%)	Liu et al. (2018)
Heterotaxy and heterotaxy-spectrum congenital heart disease	Arabic 2, Asian 2, Black or African American 13, Caucasian 90, mixed 8, unknown 42, and Hispanic/Latino 68	Array-based genotyping methods	CNVs identified in 20% of cases, total 225 patients were included in the study	Cowan et al. (2016)
Heterotaxy patients	European ancestry 120, Hispanic 104, African Americans 19, and all other ancestries 19	Genotyping using, Illumina 610Quad Bead chip platform	45 previously unrecorded CNVs in 39 different subjects, total 262 cases were included in the study (14.8%)	Fakhro et al. (2011)
Situs inversus totalis, heterotaxy, and congenital heart disease	Chinese	Whole-exome and genome sequencing of family trios	4/61 families (6.5%)	Chen et al. (2019)
Laterality defects	White non-Hispanic 158, Hispanic 109, African American 22, East Asian 10, Mediterranean 2, mixed 6, and unknown*16	Whole-exome sequencing	25/323 cases (7.7%)	Li et al. (2019)
15 situs inversus patients (6 with PCD and 9 without PCD)	Ethnicity information not available	Genome sequencing of 15 situs inversus cases and 15 controls to identify rare, highly penetrant variants in non-PCD situs inversus group	10/15 cases (66.6%)	Postema et al. (2020)
Primary ciliary dyskinesia	Dutch	Targeted-exome panel of 310 genes	50/74 cases (67.5%)	Paff et al. (2018)
Primary ciliary dyskinesia	Mixed (White, Sri Lankan, Portuguese, Hispanic, Pakistani, and Somali)	Sanger sequencing of 12 PCD-associated genes, followed by analysis of negative and single variant cases by targeted Copy Number Variation (CNV) and/or whole-exome sequencing	34/45 families (75.5%)	Marshall et al. (2015)
Suspected primary ciliary dyskinesia	Egyptian	Targeted-exome panel of 321 genes	23/33 families (69.6%)	Fassad et al. (2020b)
Primary ciliary dyskinesia	Chinese	Whole-exome sequencing	51/75 cases (68%)	Guan et al. (2021)
36 cases primary ciliary dyskinesia, 8 cases without PCD (not diagnosed as PCD), and 4 cases with inconclusive diagnosis	Jewish 13, Arabic 35	Whole-exome sequencing	34/36 PCD cases (94.4%); 4/8 (50%) cases without PCD and none of the 4 cases with inconclusive diagnosis was solved	Gileles-Hillel et al. (2020)
Primary ciliary dyskinesia	European 74, South Asian 35, Arabic 29, and all other ancestries 23	Targeted NGS panel	132/161 families (82%)	Fassad et al. (2020a)
Primary ciliary dyskinesia	Turkish	Whole-exome sequencing	46/265 cases (17.3%)	Emiralioglu et al. (2020)
Primary ciliary dyskinesia	Unknown	Candidate gene screening or whole-exome sequencing	68/75 cases (90.6%)	Blanchon et al. (2020)
Suspected primary ciliary dyskinesia	Serbian	Clinical exome panel of 29 genes	9/21 cases (42.8%)	Andjelkovic et al. (2018)
Suspected primary ciliary dyskinesia	Arabic	Clinical exome sequencing	38/56 families (67.8%)	Shamseldin et al. (2020)
Suspected primary ciliary dyskinesia	Tunisian	Targeted NGS panel of 40 PCD genes	28/34 families (82.3%)	Mani et al. (2020)
Primary ciliary dyskinesia	Samples collected from the Czech Republic, ethnicity not mentioned	Targeted NGS panel and Sanger sequencing	22/33 families (66.6%)	Djakow et al. (2016)
Suspected primary ciliary dyskinesia	Japanese	Targeted NGS panel of 32 genes	10/46 cases (21.7%)	Takeuchi et al. (2018)

## Materials and Methods

### DNA Samples

Inclusion criteria were the presence of situs inversus totalis, thoracalis or abdominalis, or left or right isomerism with or without the presence of structural heart defects. The majority of cases were children younger than 10 years, presenting to the local genetics clinic after a referral from local pediatricians or pediatric cardiologists, while the remaining cases were fetal cases diagnosed by prenatal ultrasound. None of the included children was reported by the parents to present with daily wet cough or chronic rhinitis. Informed consent was obtained from all the participants or their legal guardians. Ethical approval was obtained from the local ethics committee and samples were processed at Radboudumc in Nijmegen under the diagnostic innovation program to establish a genetic diagnosis (CMO-2006/048). Genomic DNA extraction was performed from EDTA blood samples using the standard salting out method (Miller et al., 1988) or commercially available kits such as Qiagen genomic DNA extraction kit (Germantown, MD, United States). The concentration of DNA was determined using the Nanodrop (Thermo Fisher Scientific, Waltham, MA, United States) or by Qubit 2.0 (Life Technologies, Carlsbad, CA, United States).

### Whole-Exome Sequencing

Exome sequencing was performed as previously described (Loges et al., 2018). In brief, 2–5 micrograms of DNA from index cases were subjected to whole-exome sequencing (WES) at Novogene, Hong Kong. Exome capture was performed using the Agilent SureSelect Human All Exon V5 Kit; sequencing was performed using an Illumina HiSeq 2500 machine. Paired-end sequencing was performed resulting in sequences of 150 bases from each end of the fragments. UCSC hg19 was used as a reference genome. VarScan version 2.2.5 and MuTec and GATK Somatic Indel Detector were used to detect single-nucleotide variants (SNV) and InDels. Data were then filtered in-house using a minor allele frequency (MAF) < 1% in public control databases including dbSNP, ExAc, and gnomAD, and remaining variants were first filtered for known disease-causing genes with an emphasis on diseases compatible with the patient phenotype (laterality disorder) and genes causing laterality defects in animal models. We then filtered genes encoding for proteins present in the cilia proteome and subsequently used a list of genes expressed highly in the mouse embryonic node (*S. J. Arnold*, *unpublished data*) for further gene prioritization. A complete list of genes used for filtering is shown in Supplementary Table S1. Open exome analysis was subsequently performed for cases where no likely causative allele could be detected. We prioritized homozygous variants in known consanguineous families; however, compound heterozygosity or dominant inheritance was likewise considered. Additionally, visual BAM file inspection was performed for homozygous CNVs in genes previously associated with laterality defects, and exome data files were further analyzed for the presence of CNVs using ExomeDepth (Plagnol et al., 2012).

### PCR and Sanger Sequencing

To confirm deletions identified by CNV analysis, respective exons (*DNAI2* exons 8–13 and *CCDC40* exon*s* 1–3) were amplified using the one taq master mix following the manufacturer’s instructions (New England Biolabs, Massachusetts, United States). Primers were designed using the primer blast tool, and sequences of the primers are available on request.

## Results

We performed genetic diagnostics using exome sequencing in 37 individuals, from 35 unrelated Turkish families, presenting with laterality defects to local genetics clinics. The included cases neither had a clinical history of PCD nor had an official diagnosis of PCD; however, airway ciliary motility assessment or nasal nitric oxide (NO) measurement was not performed prior to genetic investigations. Exome sequencing and CNV analysis identified likely causative variants in 14 families and candidate variants of unknown significance in 3 families, with the majority of mutations found in genes encoding for ciliary proteins of which in turn most encode for components of the ciliary motility apparatus such as dynein arms (DNAH5, DNAI1, DNAI2, DNAH9, and DNAH6), dynein arm-docking complex components (CCDC114), dynein assembly complex components (DNAAF3) or 96 nm ruler components (CCDC39 and CCDC40), and MNS1, a protein interacting with CCDC114 *in vitro* (Table 2). The vast majority of identified variants were found in a homozygous state which reflects the mainly autosomal recessive inheritance pattern seen in ciliopathies and the consanguineous nature of our subject population. In accordance with previous findings in mucociliary clearance disorders, we identified mainly nonsense, frameshift, or splice site variants (Fassad et al., 2020a). Of all causative variants identified, 8 variants have not been previously reported as disease-causing. In three additional families, we identified potential candidate disease variants in *GJA1* in SI-18, in *DNAH6* in SI-15, and in *CCDC39* and *ACVR2B* in SI-47 (Table 2).

**TABLE 2:** Genetic variants identified by exome sequencing in 35 laterality defect families.

Case	Phenotype	Consanguinity	Gene	cDNA position; dbSNP	Protein position	ClinVar/published	gnomAD allele frequency	Protein function
SI-2	*Situs inversus totalis*	Yes	*MNS1*	c.724C > T, rs185005213	p.Arg242*	Pathogenic (Ta-Shma et al., 2018)	0.0002366	Outer dynein arm docking complex
			NM_018365.4	Case previously published in Ta-Shma et al. (2018)	Homozygous			
SI-3	Situs inversus totalis	Yes	*CCDC114*	c.1004-1005 del	p.Phe335Cysfs*2	Not reported	Not reported	Outer dynein arm docking complex
			NM_001364171.2		Homozygous			
SI-7	Situs inversus totalis	Yes	*DNAAF3*	c. 1352T > C	p.Phe451Ser	Not reported	Not reported	Dynein arm assembly factor
			NM_001256715.2		Homozygous			
SI-8	Situs inversus totalis	No	*PKD1L1*	c. 7663C > T rs200853469	p.Arg2555*, heterozygous	Not reported	0.00005907	Ciliary calcium channel
			NM_138295.5	c.7937C > G, rs752673990	p.Ser2646*, heterozygous (compound heterozygous)	Not reported (Berauer et al., 2019)	0.000003977	
SI-9	Situs inversus totalis	Yes	*DNAI2*	c. 787C > T rs137852998	p.Arg263*	Pathogenic (Loges et al., 2008)	0.00001774	Outer dynein arm intermediate chain
			NM_023036.6		Homozygous			
SI-16	Situs inversus totalis	Yes	*DNAI2*	CNV, c.1212_1818del^#,+^, Exon10-13 deletion	Homozygous	Not reported	Not reported	Outer dynein arm intermediate chain
			NM_023036.6					
SI-17	Situs inversus totalis	Yes	*DNAH5*	c. 6037C > T, rs1273352530	p.Arg 2013*	Pathogenic/likely pathogenic​ (Hornef et al., 2006)	NA	Outer dynein arm heavy chain
			NM_001369.3		Homozygous			
SI-19	Situs inversus totalis	Yes	*CCDC40*	CNV: c.1_93del^#,+^	Homozygous	Not reported	Not reported	Cilia 96-nm Axonemal ruler
			NM_017950.4	Exon1-2 deletion				
SI-21	Situs inversus totalis	Yes	*CCDC114*	c.1502+5G > A, rs201133219	p.Ser469Argfs*7 homozygous	Pathogenic/likely pathogenic​ (Knowles et al., 2013)	0.00007827	Outer dynein arm docking complex
			NM_001364171.2					
SI-27	*Situs inversus totalis*		CCDC114	c. 1244T > A, rs748335075	p.Ile415Asn	Not reported	0.000003986	Outer dynein arm docking complex
			NM_001364171.2		Homozygous			
SI-41	Situs inversus totalis	No	*PIFO*	c. 239G > A^#^, rs150508940	p.Arg80Lys, heterozygous	Not reported (Kinzel et al., 2010)	0.0002829	Regulates primary cilia disassembly, localized at basal body and ciliary necklace
			NM_181643.6					
SI-44	Situs inversus totalis	Yes	*DNAI1*	c. 1333_1334insC ^#^	p.Met445Thrfs*6	Not reported	Not reported	Outer dynein arm intermediate chain
			NM_012144.4		Homozygous			
SI_45	Situs inversus totalis	Yes	*DNAH5*	c. 9346C > T^#^ rs1264701182	p.Arg3116*	Pathogenic​	0.00001992	Outer dynein arm heavy chain
			NM_001369.3		Homozygous			
SI-46	Situs inversus totalis	Yes	*DNAH9*	c.10127dupT	p.Leu3376Phefs*57 homozygous	Pathogenic​ (Loges et al., 2018)	Not reported	Outer dynein arm heavy chain
			NM_001372.4	rs867177356				
				Case previously published in Loges et al. (2018)				
SI-18	Situs inversus totalis	No	*GJA1*	c. 1001C > T, rs1460872904,	p.Pro334Leu, heterozygous, *de novo*	Not reported	0.000007962	Gap junctions
			NM_000165.5					
SI-15	Situs inversus totalis	?	*DNAH6*	c. 1020C > A	p.Tyr340* heterozygous	Not reported	Not reported	Inner dynein arm heavy chain
			NM_001370.2,	c.8829+208 C>T	p.? heterozygous	Not reported	Not reported	
SI-47	Situs inversus totalis	Yes	*CCDC39*	c. 350A > G ^*#*^	p.Asp117Gly	Uncertain significance​	Not reported	Cilia 96-nm Axonemal ruler
			NM_181426	rs1560092712	Homozygous			
			*ACVR2B*	c. 925C > T ^*#*^	p.Arg309Cys	Uncertain significance​	0.00001770	Gonadal polypeptide hormones
			NM_001106		Heterozygous			

*#*no segregation analysis performed; + intronic breakpoints not defined.

### Variants Identified in Genes Encoding for Ciliary Motility Component-Related Genes

In total, 12 out of 15 likely disease-causing variants were identified in genes encoding for ciliary motility related proteins associated with axonemal dynein motor, including two nonsense *DNAH5* variants in SI-17 and SI-45: p.Arg 2013* previously published by Hornef et al. (2006) and p.Arg3116* not previously reported as disease-causing. In SI-46, we identified a homozygous frameshift variant in *DNAH9*, p.Leu3376Phefs*57 (case and mutation meanwhile reported by Loges et al. (2018).

We further found three families to carry variants in genes encoding for dynein intermediate chains: *DNAI1* c.1333_1334insC, p.Met445Thrfs*6 not previously reported as disease-causing or in gnomAD for SI-44 and *DNAI2* p.Arg263* previously identified by Loges et al. in SI-9 (Loges et al., 2008) and a homozygous CNV encompassing *DNAI2* exons 10–13 (c.1212_1818del) in SI-16 not previously reported and confirmed by PCR (Supplementary Figures S1, S2). Our analysis further revealed 3 homozygous variants in the outer dynein arm-docking complex (ODA-DC) component *CCDC114:* in SI-3, we identified c.1004-1005del, p.Phe335Cysfs*2 not previously reported, while in SI-21, we detected the previously reported splicing variant, c.1502+5G > A (Knowles et al., 2013) and a not previously reported homozygous missense, p.Ile415Asn in SI-27. Knowles et al. found that the splice site change in intron 12 results in a frameshift and introduces a stop codon (p.Ser469Argfs^*^7). The homozygous missense change p.Ile415Asn in SI-27 is not reported in ClinVar, predicted deleterious by SIFT, probably damaging (0.997) by PolyPhen and polymorphism by mutation taster. The mutant residue is bigger, and this will change the 3D structure and might lead to bumps. The different hydrophobicity of the wild-type and mutant residues could cause a loss of protein–protein interactions (mutation prediction program used: HOPE; https://www3.cmbi.umcn.nl/hope/). Segregation analysis revealed the mother to be a heterozygous carrier; however, the variant was not detected in the father. This could be due to a heterozygous not detected deletion on the other allele, non-paternity, *de novo* occurrence of the mutation in the child or maternal uniparental disomy. In SI-2, we identified a homozygous nonsense change (p.Arg242*) in *MNS1* (case and variant reported in Ta-Shma et al., 2018 (Ta-Shma et al., 2018). Interestingly, this variant was identified in several unrelated individuals with laterality defects and perturbed male fertility but no respiratory symptoms (Ta-Shma et al., 2018). MNS1 interacts with CCDC114, suggesting likewise a role for outer dynein arm docking (Ta-Shma et al., 2018). In SI-7, we identified a not previously reported homozygous missense variant, p.Phe451Ser in *DNAAF3.* The variant is probably damaged (0.998) by PolyPhen prediction and polymorphism using mutation taster analysis. The wild-type and mutant amino acids differ in size with the mutant residue being smaller, and this might influence the protein structure and protein–protein interactions. Furthermore, due to differences in hydrophobicity of the wild-type and mutant residue, hydrophobic interactions, either in the core of the protein or on the surface, could be lost (https://www3.cmbi.umcn.nl/hope/).

CNV analysis revealed a novel homozygous deletion encompassing c.1_93 of *CCDC40* in SI-19 conformed by PCR Supplementary Figures S3, S4. We could not identify the intronic breakpoints of the CNVs identified in this study.

### Variants Identified in Other Ciliary Genes

We identified mutations in two genes encoding for ciliary proteins not related to ciliary motility, *PIFO* and *PKD1L1. PIFO* p.Arg80Lys was identified heterozygously in SI-41, previously reported by Kinzel et al. in two cases in a heterozygous state (Kinzel et al., 2010). One previously reported case represented a deceased neonate with thoracic–abdominal *situs inversus*, cystic kidneys, and liver fibrosis, and a second case suffered from isolated double-outlet right ventricle (Kinzel et al., 2010). *PIFO* has been previously identified as a gene expressed in the node by Tamplin et al. using microarray analysis in *Foxa2* mutant mouse embryos (Tamplin et al., 2008). Parental DNA was not available to confirm *de novo* occurrence of the variant in our case. *PKD1L1* p.Arg2555* and p.Ser2646* were identified as compound heterozygosity in SI-8, both not reported in ClinVar to date. Biallelic variants in *PKD1L1* have been previously reported in individuals with laterality defects and congenital heart malformations (Vetrini et al., 2016; Postema et al., 2020; Bolkier et al., 2021; Correa et al., 2021). The variant p.Ser2646* identified in our study was also reported by Berauer et al. in a case with polysplenia, biliary atresia, abdominal heterotaxy, congenital heart disease, and vascular anomalies (Berauer et al., 2019). PKD1L1 seems to form a ciliary protein complex with PKD2 in primary crown cell cilia in the node, and it thought to play a role in flow sensing (Field et al., 2011).

### Candidate Variants Identified

Candidate variants were identified in three families (SI-18, SI-15 and SI-47). SI-18 carries a heterozygous *de novo* missense variant in *GJA1* (p.Pro334Leu). The mutant residue is bigger, and this might lead to bumps. The mutation is located within a stretch of residues annotated in UniProt as a special protein–protein interaction region. The wild-type residue is a proline. Prolines are known to be very rigid and therefore induce a special backbone conformation which might be required at this position. The mutation can disturb this special conformation (https://www3.cmbi.umcn.nl/hope/). This variant is not reported in ClinVar and has a frequency of 0.000007962 in gnomad. The variant is predicted to be benign (0.020) by PolyPhen, disease-causing by mutation taster (score 98), and tolerated by SIFT prediction. The amino acid position is conserved in mammals including mice, chicken, and *Xenopus* while zebrafish carries an alanine instead here.

In SI-15, we identified a heterozygous stop variant, c.1020C > A, p.Tyr340* and a heterozygous deep-intronic substitution, c.8229+208C>T in *DNAH6*. While the Alamut program predicted only minimal splice site probabilities for the mutant allele compared to the wild-type sequence, ESE finder predicted a slightly changed pattern of ESE binding (Supplementary Figures S5, S6).

**FIGURE 3:**
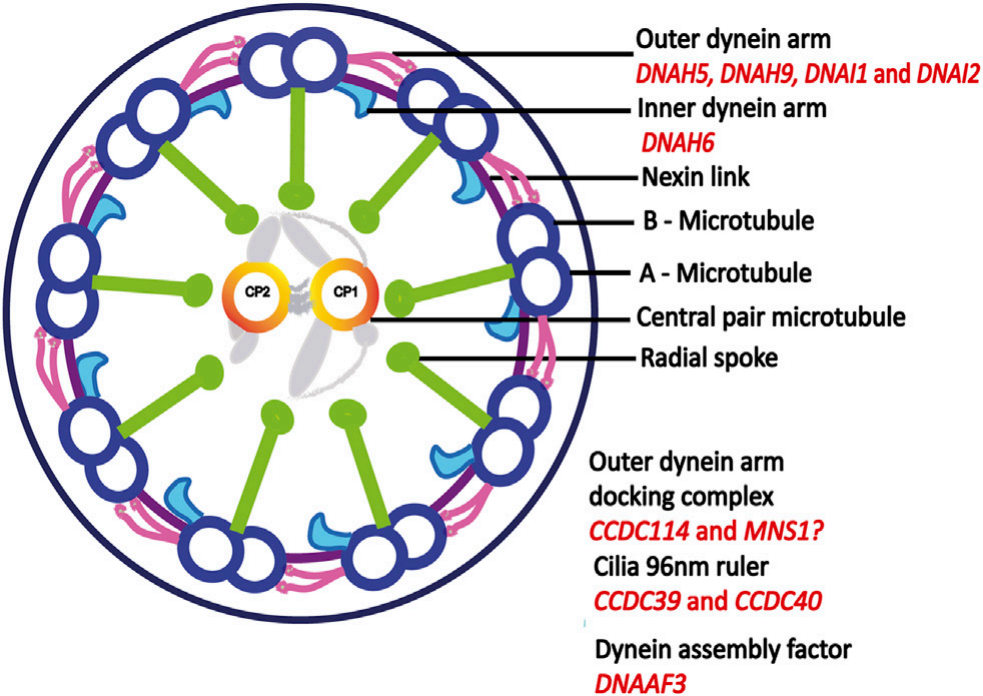
Motile cilia ultrastructure. Motile cilia are microtubule based structures, dynein arms powers the sliding motion of microtubules which in turn generates ciliary movement. Outer dynein arm-docking complex helps in the attachment of dynein arms to A-microtubule. Dynein assembly factor is important for the assembly of both outer and inner dynein arms. Mutations in central pair and radial spoke genes does not cause laterality defect, adapted from (Antony et al., 2021). Proteins encoded by genes identified in this study are indicated in red.

**FIGURE 4:**
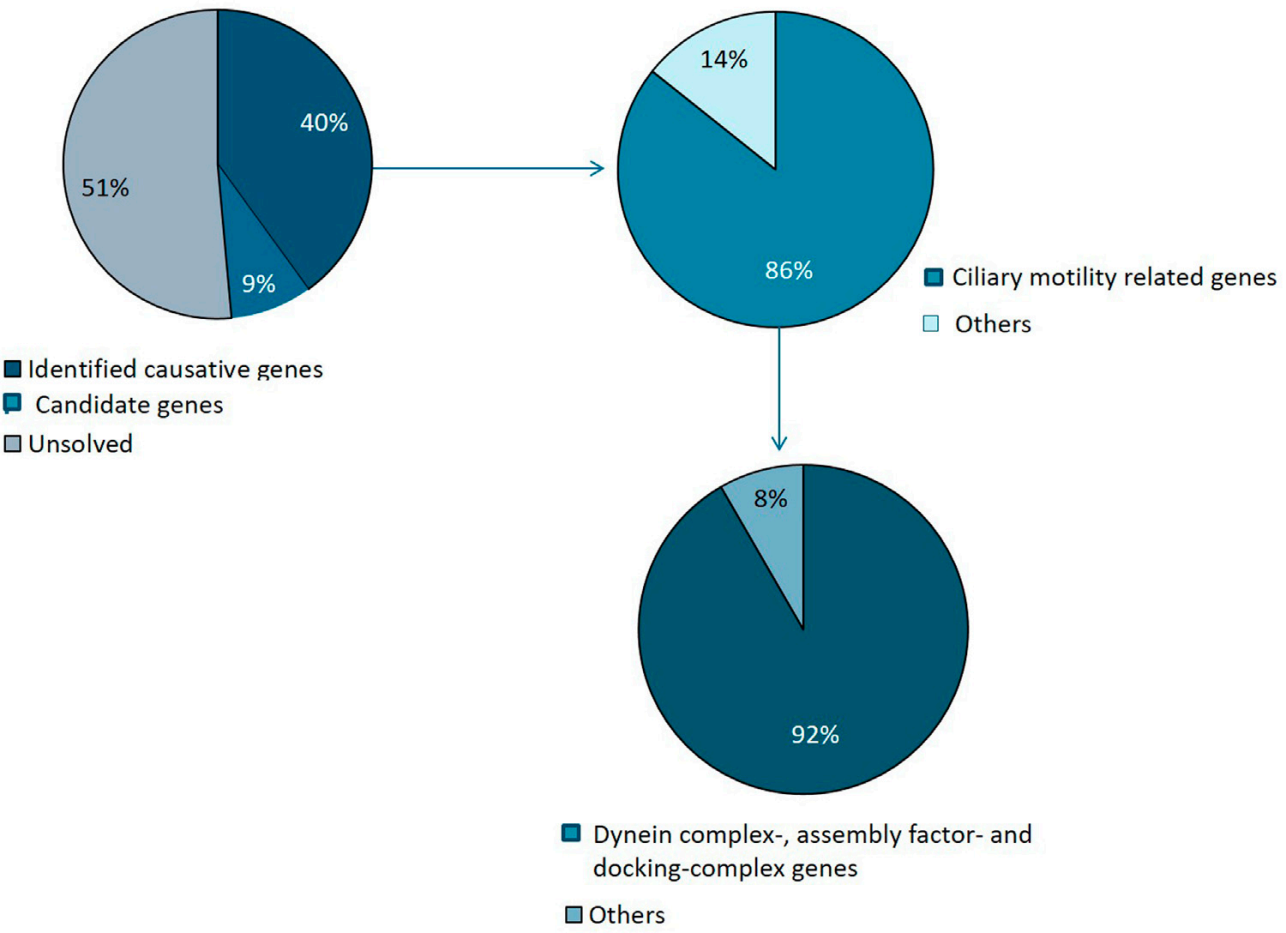
Summary of genetic variants identified. Causative mutations were identified in 14 families and candidate variants in 3 families. The majority of identified variants were in ciliary genes, mainly encoding motility apparatus components, of which the majority represents dynein complex related factors.

In SI-47, we identified variants in two genes, *CCDC39* and *ACVR2B*: a homozygous missense variant in *CCDC39* (p.Asp117Gly), not reported in gnomAD but in ClinVar as a variant of uncertain significance (VUS). The variant is predicted as polymorphism by mutation taster and PolyPhen benign with a score of 0.351. The variant is located at the first coiled–coil domain (16–122 amino acids; UniProt). Additionally, we identified a heterozygous variant in *ACVR2B,* p.Arg309Cys, predicted damaging by SIFT, probably damaging (1.000) by PolyPhen, and disease-causing by mutation taster, likewise reported as VUS in ClinVar. The variant is located in the protein kinase-binding domain (190–480 amino acids). Segregation analysis could not be performed to establish if the variant is *de novo*. Several previous studies have reported heterozygous *ACVR2B* variants in individuals with heterotaxy (Kosaki et al., 1999; Ma et al., 2012; Li et al., 2019). *ACVR2B* functions in the NODAL/TGF-beta signaling pathway (Li et al., 2019). A summary of all genetic findings is shown in Table 2 and Figures 3, 4.

## Discussion

Over the last 2 decades, genetic analyses of individuals with laterality defects have greatly advanced our understanding of establishment of left–right asymmetry. Eight variants reported here have not been previously reported as disease-causing, two of which are CNVs. This provides useful information for human genetic diagnostics and underlines the importance of CNV analysis.

Given the fact that none of the cases included in this study had a formal diagnosis of PCD nor was suspected with PCD clinically, we identified pathogenic or likely pathogenic variants in a rather large number of genes known to cause PCD when dysfunctional, including *DNAH5*, *DNAH9*, *CCDC114, DNAI1, DNAI2*, *CCDC39*, and *CCDC40*. *DNAH5* encodes an outer dynein arm γ—heavy chain distributed panaxonemally in respiratory cilia, and biallelic loss of function mutations are a frequent cause of PCD (Hornef et al., 2006). *DNAH9* encodes an outer dynein arm (ODA) heavy chain only present in one of 2 ODA subtypes, subtype 2. ODA2 localization is restricted to the distal half of the cilium and accordingly, and DNAH9 dysfunction results in a very subtle respiratory phenotype with perturbed distal ciliary movements (Loges et al., 2018). *CCDC114* mutation causes the absence of the outer dynein arm in affected individuals, and majority of the cilia are static with few showing some stiff movements (Knowles et al., 2013; Onoufriadis et al., 2013). *DNAAF3* encodes a cytoplasmic dynein preassembly factor. Dynein preassembly factors play a crucial role for the assembly of both outer and inner dynein arms and dysfunction results in cilia lacking dynein arms, causing ciliary immotility (Mitchison et al., 2012). *DNAI1* and *DNAI2* encode outer dynein arm intermediate chains and dysfunction of these genes results in the absence of outer dynein arms causing immotile cilia or cilia with minimum residual motility (Guichard et al., 2001; Loges et al., 2008). *CCDC40* encodes a 96-nm ruler protein, and CCDC40 loss of function results in a disorganized microtubule arrangement and radial spokes and nexin link defects and absence of inner dynein arms. The respiratory cilia of the individuals carrying biallelic *CCDC40* loss of function variants show defective ciliary beat regulation and ciliary movements with a reduced amplitude (Becker-Heck et al., 2011). *CCDC39* likewise encodes a 96-nm ruler protein in motile cilia and dysfunction results in disorganized microtubules, defects in radial spokes, inner dynein arms, and nexin links, similar to what is observed with dysfunction of CCDC40 (Merveille et al., 2011). Our findings confirm genetic PCD studies showing that *DNAH5* and *CCDC40* mutations represent common underlying causes (Fassad et al., 2020a).

We further identified candidate variants in *DNAH6* in *SI-15*. In humans, *DNAH6* encodes a heavy chain of the inner dynein arm (Hom et al., 2011). Li et al. suggested that *DNAH6* mutations play a role in laterality defects (Li et al., 2016) when they reported an individual with heterotaxy carrying a heterozygous *DNAH6* mutation and a heterozygous *DNAI1* mutation, suggesting digenic inheritance/genetic interaction. Moreover, 5/6 patients in their study with heterozygous *DNAH6* mutations also harbored heterozygous *DNAH5* mutations. The subthreshold double-morpholino knockdown of *DNAH6*/*DNAH5* and *DNAH6*/*DNAI1* in zebrafish resulted in likewise heterotaxy. Re-analyzing the exome data for potentially causative heterozygous mutations in *DNAI1* and *DNAH5* in SI-15 did not yield any new causative variants. Compound heterozygous mutations in *DNAH6* are also reported in patients with sperm flagella defects (Li et al., 2018; Tu et al., 2019).

The *PIFO* heterozygous variant we identified was previously reported to cause laterality defects in a single individual of Northern European descent (Kinzel et al., 2010). PIFO plays an important role in primary cilia disassembly through AuraA activation and it is localized at the basal body (Kinzel et al., 2010). The p.Arf80Lys variant seemed to inhibit AurA activation. Furthermore, overexpression caused cilia disassembly defects (Kinzel et al., 2010). In contrast to the previously described case, no liver or kidney cysts have been observed in our case to date; however, later development in life cannot be excluded and appropriate clinical monitoring has been initiated. Our study confirms that the identified *PIFO* variant is a rare cause of left–right pattern defects in humans, and it seems likely that future studies will reveal more disease-causing *PIFO* alleles.

PKD1L1 is a subunit of the heterodimeric TRP channel thought to regulate calcium currents (Delling et al., 2013) and is important for confining the NODAL expression to the left side during the left–right axis determination (Grimes et al., 2016). The PKD1L1 p.Arg2555* and p.Ser2646* variants we identified in this study are located at the extracellular domains before the trans-membrane domains 8 and 10 respectively (Rodriguez et al., 2019; Correa et al., 2021). These two compound heterozygous changes identified in this study could affect the function of trans-membrane domains 8–10, extracellular domains between them, and an intracellular coiled–coil domain, which interacts with PKD2. The PKD1L1 variants are similarly identified in other laterality defect cases at N-terminal extracellular domains preceding immunoglobulin-like PKD, REJ (receptor egg jelly), GPR (G-protein-coupled receptor proteolytic site), trans-membrane, and extracellular domains in the middle of the trans-membrane domains and intracellular C-terminal coiled–coil domain (Berauer et al., 2019; Rodriguez et al., 2019; Correa et al., 2021).

The association of *GJA1* with laterality defects or congenital heart disease remains unsure. *GJA1* encodes connexion 43 and is important for the formation of gap junction and inter cellular channels (Britz-Cunningham et al., 1995). Gap junctions could play an important role in the transfer of signals from the node to the lateral plate mesoderm in vertebrates (Saund et al., 2012). Britz-Cunningham et al. reported potentially disease-causing missense variants in *GJA1* in 6 patients with complex cardiac malformations and laterality defects (Britz-Cunningham et al., 1995). However, two later studies were not able to replicate these findings (Casey and Ballabio, 1995; Splitt et al., 1995). In 2001, Dasgupta et al. suggested that the inability to replicate the findings of the initial study could be caused by a PCR amplification bias toward the wild-type allele. Dasgupta et al. therefore used denaturing gradient gel electrophoresis (DGGE), followed by separate sequencing of two alleles and detected two heterozygous missense variants and 2 synonymous variants in eight individuals with hypoplastic left heart syndrome and one individual with atrioventricular canal defect. However, the four variants identified were identical to the pseudogene sequence at the nucleotide level (97% homology) (Dasgupta et al., 2001). Mutations in *GJA1* have further been identified in cases with autosomal recessive and dominant oculodentodigital dysplasia, where cardiac anomalies are rare (OMIM: 257850, 164200). Clinically, our cases did not exhibit features of oculodentodigital dysplasia, where about 50% of all cases represent *de novo* alleles.

Huang et al. could not confirm that *GJA1* dysfunction causes heart defects or left–right patterning defects in mice nor did they identify *GJA1* disease-causing variants in a cohort of 300 patients with congenital heart disease (Britz-Cunningham et al., 1995; Dasgupta et al., 2001; Huang et al., 2011). However, in a sudden infant death case, a *de novo* mutation in *GJA1* was detected and the patient tissue also demonstrated a mosaic expression of connexin43 (*GJA1*) (Van Norstrand et al., 2012). To our knowledge, no human cases with a laterality defect and a dominant *GJA1* *de novo* variant have been described in the literature. The novel *GJA1* variant we identified (p.Pro334Leu) was located in the *UBQLN4* (ubiquilin 4)-binding region. The interaction of *GJA1* with this ubiquitin-like and ubiquitin-associated domain-containing protein is important for its homeostasis and function (Su et al., 2010). Putatively, the mutation could influence the *GJA1*–UBQLN4 interaction.

The variant of unknown significance in *ACVR2B* we identified in SI-47 represents the only variant we identified in a gene encoding for a nodal signaling pathway component. To date, less than ten cases with *ACVR2B* dominant variants have been reported (Kosaki et al., 1999; Ma et al., 2012; Li et al., 2019). Among those, the same variant (p.Arg40His) has been identified in four cases. Generally, mutations in *ACRV2B* seem to represent a rare cause of laterality defects (Ma et al., 2012). As we also identified a VUS in *CCDC39* in SI-47, the role of the *ACRV2B* variant we identified remains unclear.

To date, only few studies investigating cohorts of cases with laterality defects have been published and those have generally exhibited low mutation detection rates (Table 1), including a large study identifying pathogenic variants in just 25/323 cases (Li et al., 2019). Among non-PCD cases, mutations in *ZIC3* and *NODAL* appear to be the most common genetic causes (Li et al., 2019). In our cohort, the rate of detected likely disease-causing variants was somewhat higher (17 of 35 families (49%), potentially reflecting the large proportion of not previously clinically identified PCD cases and a high rate of consanguineous families in our series. The patients in our study were neither selected for the presence of additional symptoms indicating PCD nor tested for motile cilia dysfunction, but still we identified the majority of causative mutations in genes associated with the ciliary motility apparatus. This is in line with previous studies where 42% of congenital heart disease patients with heterotaxy defects that had a genetic cause were found to carry mutations in mucociliary clearance disorder genes (Nakhleh et al., 2012) as well as with a mouse recessive forward genetics screen for congenital heart defects (Li et al., 2015). Furthermore, mutation detection rates are higher among human PCD cases than isolated laterality defect cases (Table 1). Overall, dysfunction of proteins associated with ciliary motility seems to be responsible for the majority of human laterality defects where a monogenetic cause can be identified with the currently used genetic diagnostics methods (Table 1). Confirming previous studies, we find that mutations in genes encoding for dynein motor complex components make up a large proportion of identified genetic variants in genes associated with ciliary motility. None of the cases in our study carrying mutations in genes associated with PCD had previously been diagnosed with PCD underlines the importance of genetics for PCD diagnostics, especially when specialized PCD diagnostics are unavailable locally, as the diagnosis of PCD allows appropriate clinical management which can significantly alter the disease course (Storm van’s Gravesande and Omran, 2005; Davis et al., 2019).

## Conclusion

Our study provides a comprehensive list of laterality defect candidate genes based on human and animal studies. Future studies are needed to further validate candidate genes in regulating laterality patterning. In light of our findings, motile ciliary genes could be prioritized in laterality defect genetic screening studies. The overall low mutation detection rate in our laterality defect cohort (49%) and previous reports (Table 1) suggests the existence of non-coding monogenetic variants, genetic mosaicism within embryonic tissues or presence of epigenetic or environmental and non-genetic factors playing a significant role(s) in defective left-right patterning.

## Data Availability

The datasets for this article are not publicly available due to concerns regarding participant/patient anonymity. Requests to access the datasets should be directed to the corresponding author.
