# Supplementary material for: Spectrum of Genetic Variants in a Cohort of 37 Laterality Defect Cases
Source: Front Genet. 2022 Apr 13;13:861236. doi: 10.3389/fgene.2022.861236 (PMC9083912; doi:10.3389/fgene.2022.861236)
Supplement: Supplementary file 2 [file DataSheet1.docx]

**Supplementary Figures**

**
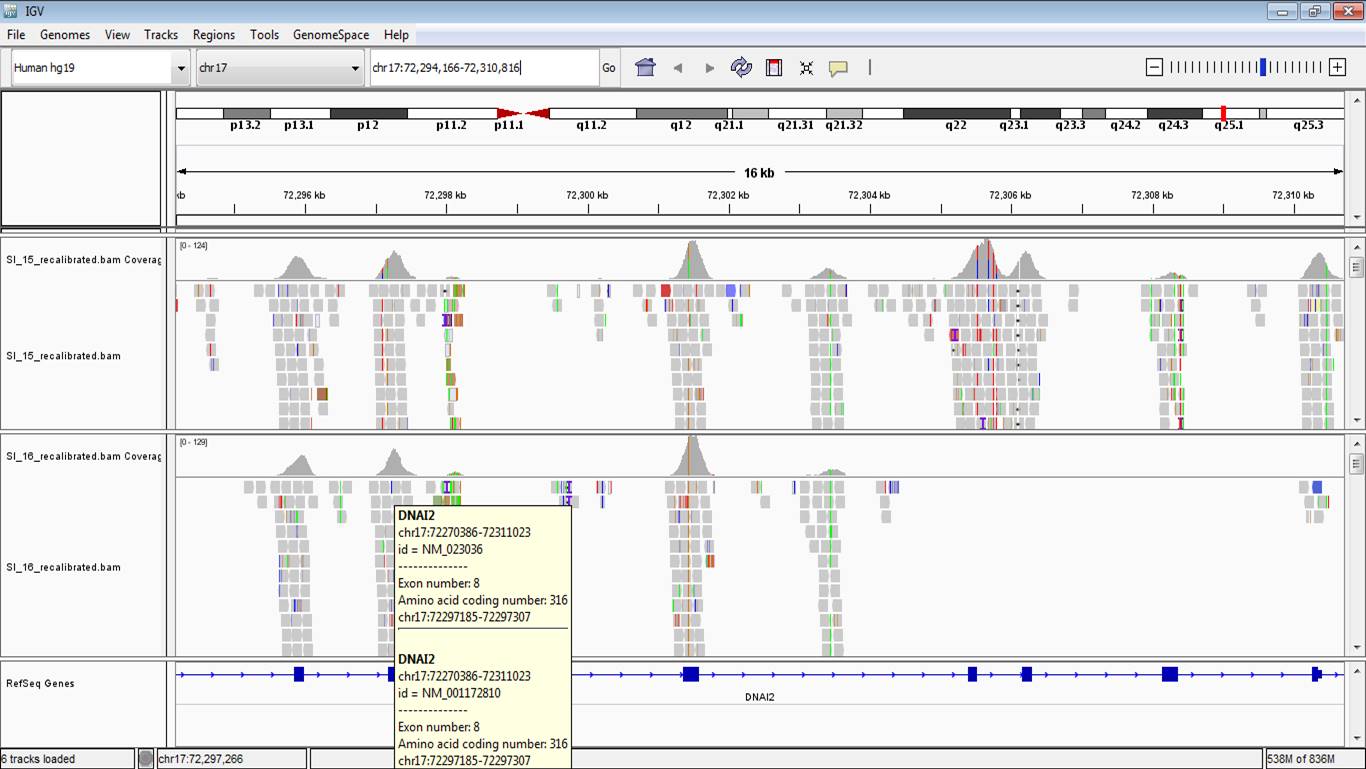
**

**Figure S1|** Screenshots from IGV showing deletion of *DNAI2*, exons 10-13 in SI-16.


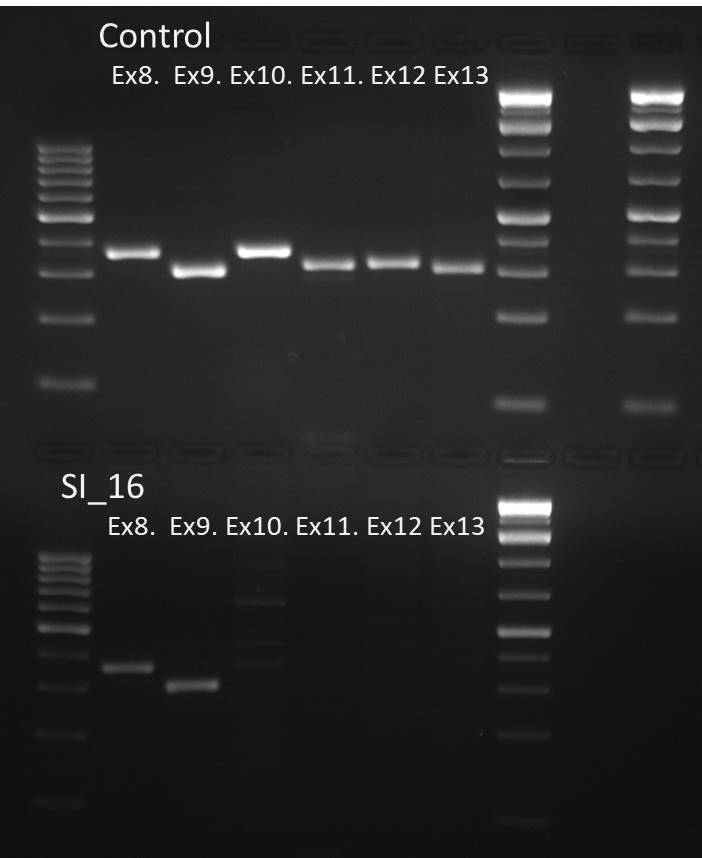


**Figure S2** PCR amplification of *DNAI2* exons 8-13 in a control sample and SI-16 showing *DNAI2* exon 10-13 deletion in SI-16 while exon 8 and 9 are normally amplified.


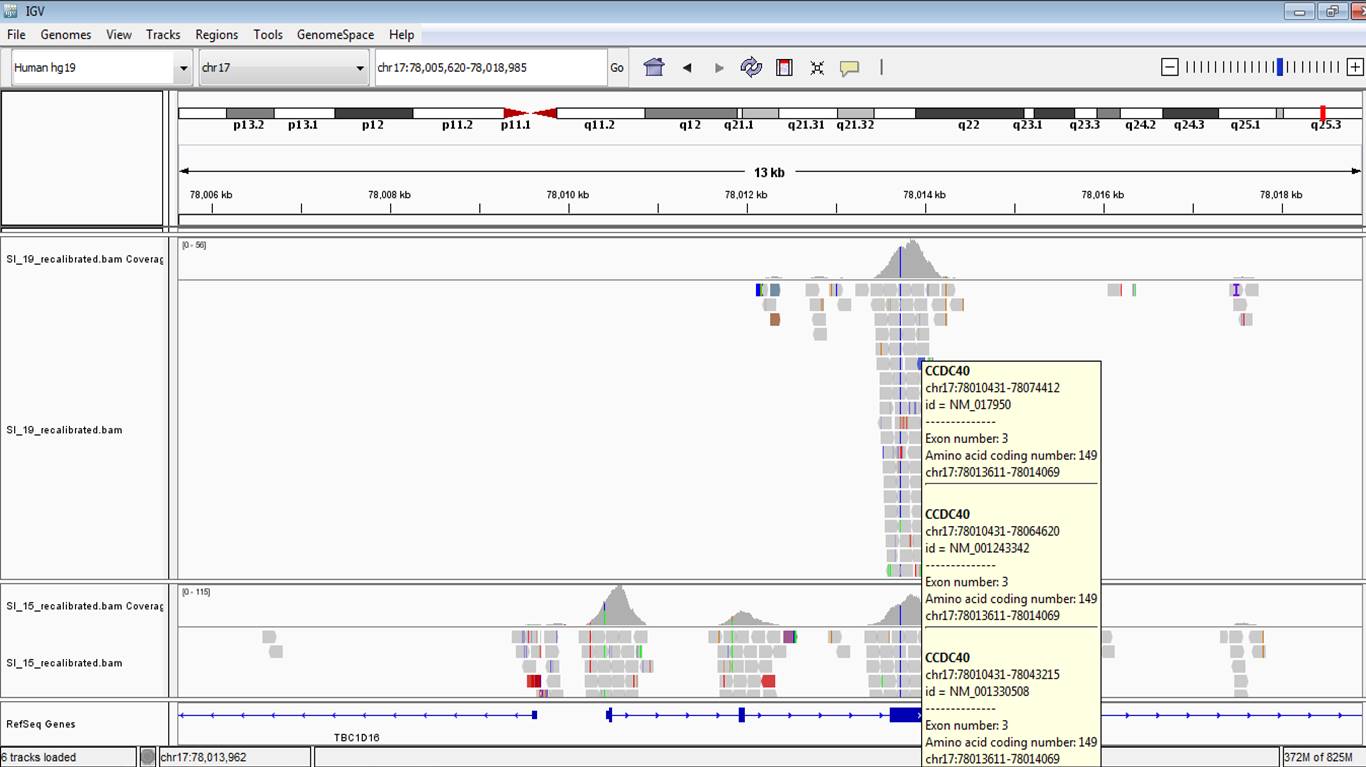


**Figure S3|** Screenshots from IGV showing deletion of *CCDC40,* exons 1-2 in SI-19.


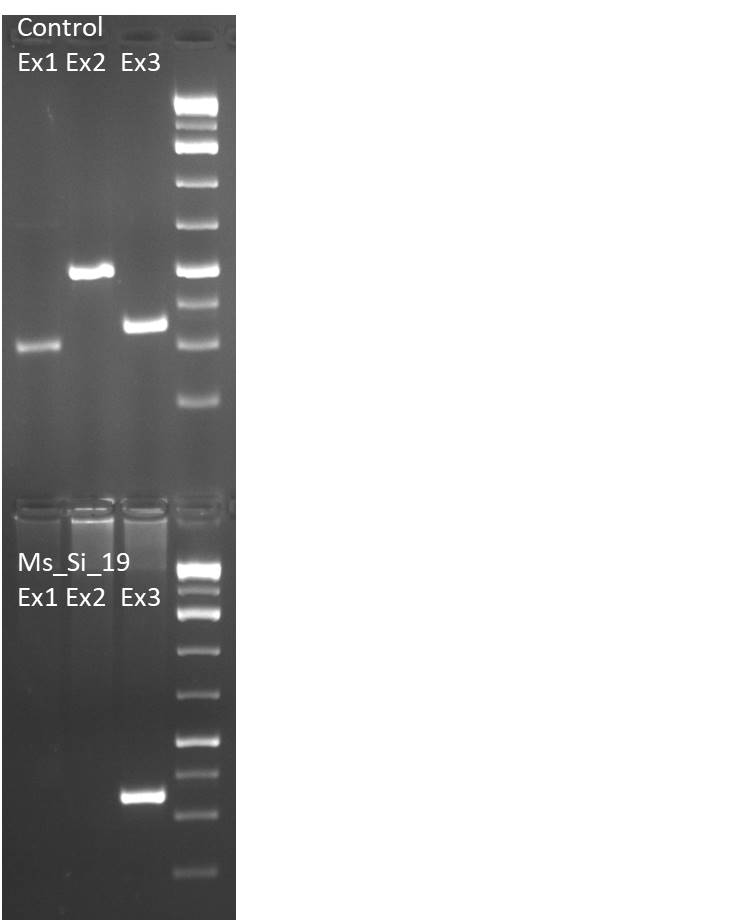


**Figure S 4|** PCR amplification of *CCDC40*, exons 1-3 in control and SI-19. In SI-19 CCDC40 exons 1 and 2 are deleted while exon 3 is normally amplified.


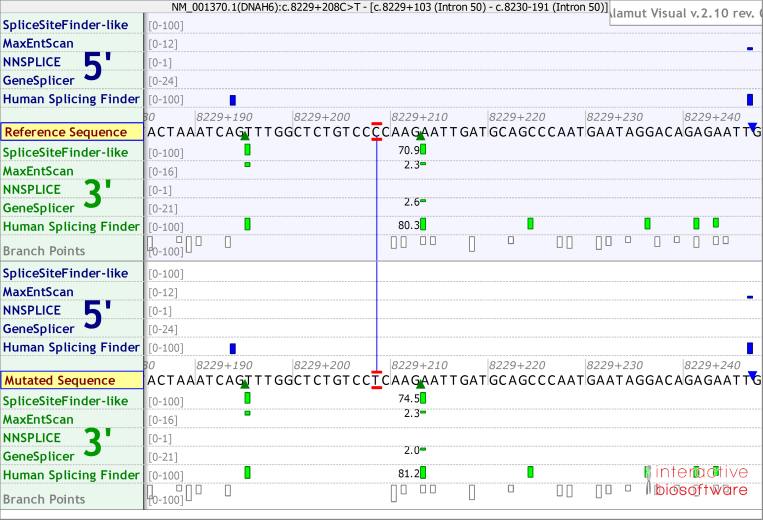


**Figure S5|** Splice prediction using Alamut visual integrated splicing predictor tools do not yield major differences between the reference and the mutated sequence concerning splicing predictor scores


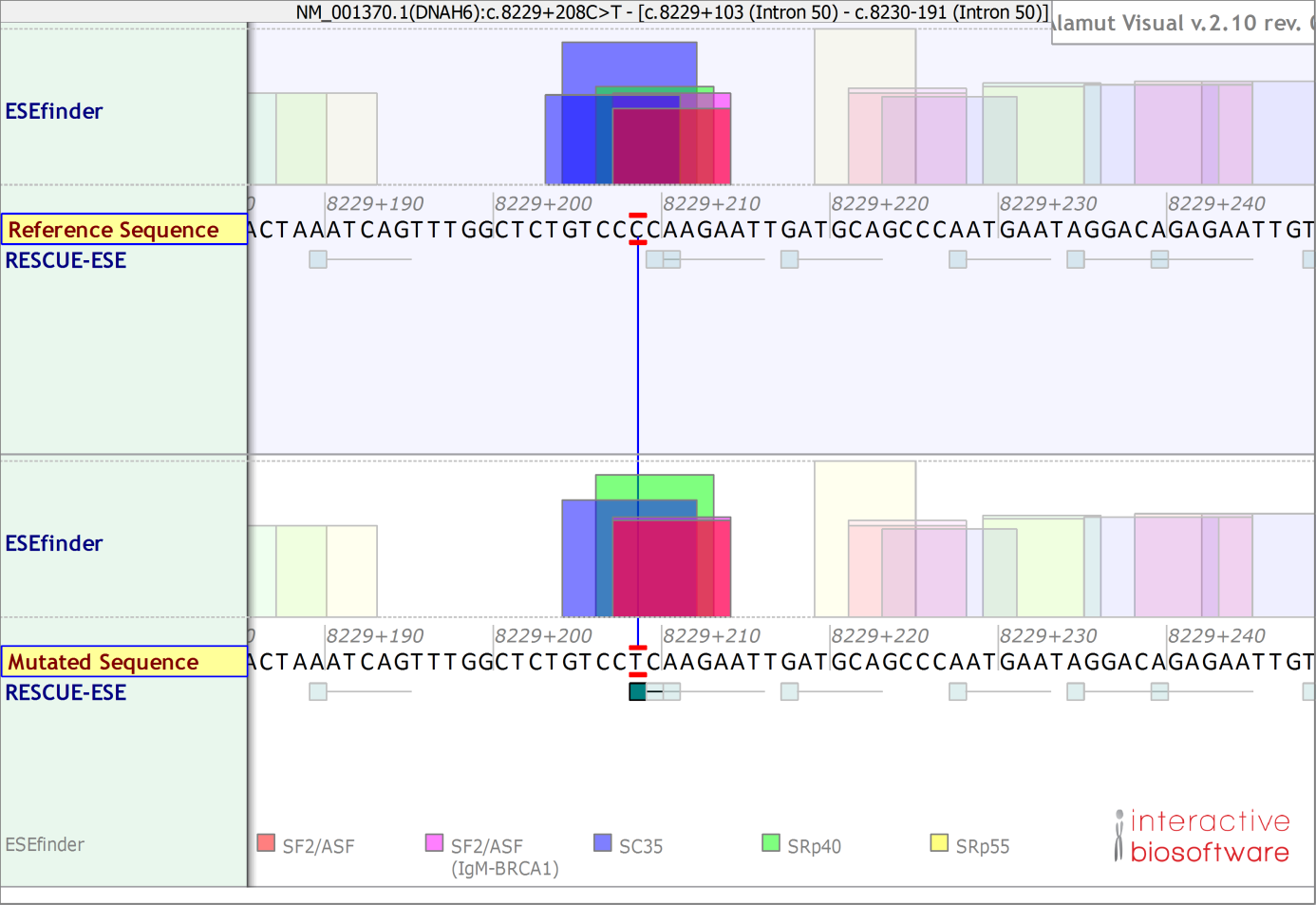


**Figure S6|** Exonic Splicing Enhancer prediction showing slight changes in the ESE pattern for c.8229+208C>T compared to wildtype sequence.
